# The Role of Tyr-His-Trp Triad and Water Molecule Near the N1-Atom of 2-Hydroperoxycoelenterazine in Bioluminescence of Hydromedusan Photoproteins: Structural and Mutagenesis Study

**DOI:** 10.3390/ijms24076869

**Published:** 2023-04-06

**Authors:** Pavel V. Natashin, Ludmila P. Burakova, Margarita I. Kovaleva, Mikhail B. Shevtsov, Daria A. Dmitrieva, Elena V. Eremeeva, Svetlana V. Markova, Alexey V. Mishin, Valentin I. Borshchevskiy, Eugene S. Vysotski

**Affiliations:** 1 Photobiology Laboratory, Institute of Biophysics of Siberian Branch of the Russian Academy of Sciences, Federal Research Center “Krasnoyarsk Science Center” of Siberian Branch of the Russian Academy of Sciences”, Krasnoyarsk 660036, Russia; pavelnatashin@mail.ru (P.V.N.); burakoval@mail.ru (L.P.B.); l_eremeeva@mail.ru (E.V.E.); smarkova@mail.ru (S.V.M.); 2 Institute of Fundamental Biology and Biotechnology, Siberian Federal University, Krasnoyarsk 660041, Russia; 3 Research Center for Molecular Mechanisms of Aging and Age-Related Diseases, Moscow Institute of Physics and Technology, Dolgoprudny 141701, Russia; kovaleva.margo@gmail.com (M.I.K.); mishevtsov@gmail.com (M.B.S.); dmitrieva.da@phystech.edu (D.A.D.); mishinalexey@phystech.edu (A.V.M.); 4 Joint Institute for Nuclear Research, Dubna 141980, Russia

**Keywords:** photoprotein, obelin, aequorin, coelenterazine, photoprotein mutants, crystal structure

## Abstract

Hydromedusan photoproteins responsible for the bioluminescence of a variety of marine jellyfish and hydroids are a unique biochemical system recognized as a stable enzyme-substrate complex consisting of apoprotein and preoxygenated coelenterazine, which is tightly bound in the protein inner cavity. The binding of calcium ions to the photoprotein molecule is only required to initiate the light emission reaction. Although numerous experimental and theoretical studies on the bioluminescence of these photoproteins were performed, many features of their functioning are yet unclear. In particular, which ionic state of dioxetanone intermediate decomposes to yield a coelenteramide in an excited state and the role of the water molecule residing in a proximity to the N1 atom of 2-hydroperoxycoelenterazine in the bioluminescence reaction are still under discussion. With the aim to elucidate the function of this water molecule as well as to pinpoint the amino acid residues presumably involved in the protonation of the primarily formed dioxetanone anion, we constructed a set of single and double obelin and aequorin mutants with substitutions of His, Trp, Tyr, and Ser to residues with different properties of side chains and investigated their bioluminescence properties (specific activity, bioluminescence spectra, stopped-flow kinetics, and fluorescence spectra of Ca^2+^-discharged photoproteins). Moreover, we determined the spatial structure of the obelin mutant with a substitution of His64, the key residue of the presumable proton transfer, to Phe. On the ground of the bioluminescence properties of the obelin and aequorin mutants as well as the spatial structures of the obelin mutants with the replacements of His64 and Tyr138, the conclusion was made that, in fact, His residue of the Tyr-His-Trp triad and the water molecule perform the “catalytic function” by transferring the proton from solvent to the dioxetanone anion to generate its neutral ionic state in complex with water, as only the decomposition of this form of dioxetanone can provide the highest light output in the light-emitting reaction of the hydromedusan photoproteins.

## 1. Introduction

The Ca^2+^-regulated photoproteins are found in a variety of luminous marine organisms [1]. The best studied of them are aequorin and obelin, which are responsible for light emission of the jellyfish *Aequorea victoria* [1] and the hydroid *Obelia longissima* [2]. All Ca^2+^-regulated photoproteins isolated to date are small single-chain proteins with a tightly bound oxygen-activated imidazopyrazinone derivative, 2-hydroperoxycoelenterazine, serving as a substrate [1,2]. The binding of calcium ions to the photoprotein molecule initiates decarboxylation of 2-hydroperoxycoelenterazine yielding a protein-bound product, coelenteramide, in an excited state [3,4]. Relaxation of the excited coelenteramide to the ground state results in a blue light emission with spectral maxima ranging from 465 to 495 nm depending on the photoprotein source [5].

In the past 35 years, the cDNA genes encoding only a few hydromedusan photoproteins such as aequorin [6,7,8], clytin [9,10,11,12], mitrocomin [13,14], and two obelins from different Obelia species [15,16,17] were cloned. The comparison of amino acid sequences revealed a high degree of identity (~60–70%) among these proteins and the presence of the three EF-hand Ca^2+^-binding sites comprised of 12 residues [2,18] typical of proteins belonging to a large family of EF-hand Ca^2+^-binding proteins [19]. Under calcium-free conditions in the presence of O_2_ and reducing agents such as dithiothreitol or β-mercaptoethanol, the recombinant apophotoproteins can easily be converted into active photoproteins by incubation with synthetic coelenterazine [20,21].

**Figure 1:**
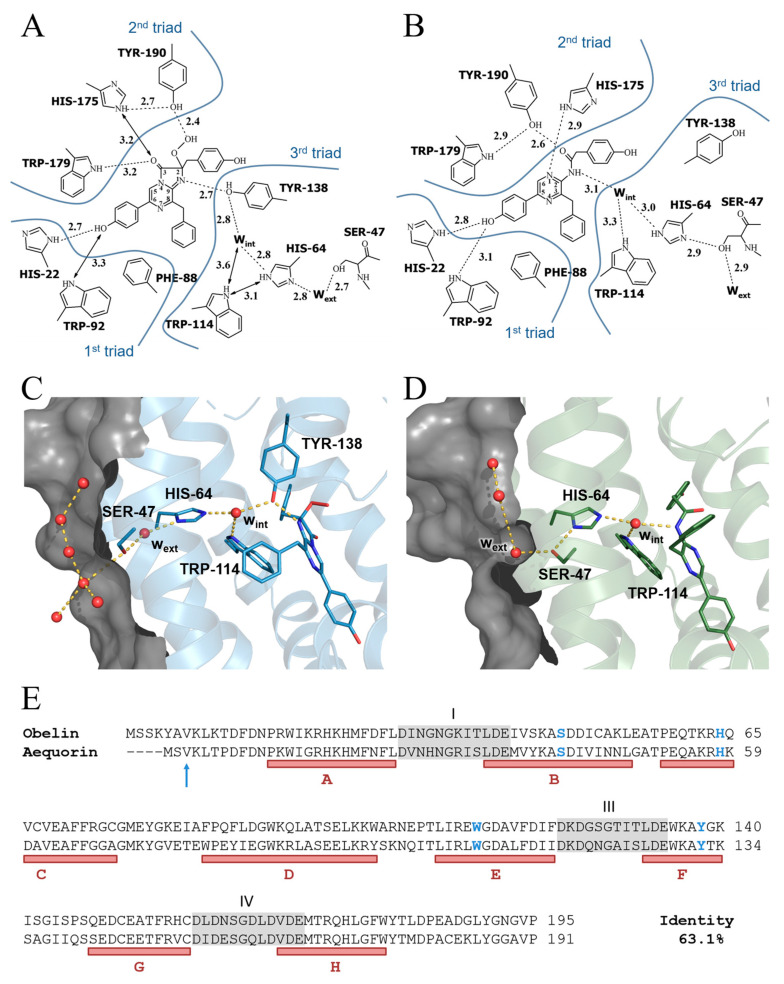
Two-dimensional drawing of the hydrogen bonding network in the binding cavities of active (**A**) and Ca^2+^-discharged (**B**) wild-type obelins. Molecular surface representation of “proton channel” in active (**C**) and Ca^2+^-discharged (**D**) wild-type obelins. Amino acid residues and the molecules of 2-hydroperoxycoelenterazine and coelenteramide are shown as stick models. Water molecules are represented as red balls. Hydrogen bonds are shown as dashed lines. Partially represented surface is gray colored. (**E**) Sequence alignment of obelin from *O. longissima* [37] and aequorin from *A. victoria* [38]. The residues in obelin and aequorin mutated in this study are shown in blue. The Ca^2+^ binding loops are highlighted in gray. The helices are indicated by capital letters A–H based on the wild-type obelin structure (PDB code 1QV0). Aequorin numeration starts from Val, shown with an arrow, according to the original numbering of the amino acid sequence determined for the natural aequorin and aequorin structure (PDB code 1EJ3) [1,22].

By now, the three-dimensional structures of the Ca^2+^-regulated photoproteins aequorin [22], obelin [23,24], clytin [25], and mitrocomin [14] have been reported. All of them exhibited compact globular structures formed by *N*- and *C*-terminal domains within the inner cavity of which the 2-hydroperoxycoelenterazine molecule is disposed. The substrate-binding pocket of these photoproteins is very hydrophobic and is formed by practically the same residues despite some differences in their sequences [22,23]. In addition to hydrophobic residues, the side chains of the three histidines and three tyrosines in the case of aequorin and mitrocomin (or two Tyr residues in obelin and clytin) are also directed into the cavity (Figure 1) [22,23]. These residues, along with tryptophans, are clustered in triads located in close proximity to the substrate molecule, suggesting the formation of hydrogen bonds with the atoms of various groups of the 2-hydroperoxy adduct of coelenterazine. The first His-Trp-Tyr (Phe instead of Tyr in obelin and clytin) triad surrounding the OH group of the 6-(*p*-hydroxyphenyl) substituent of the substrate (Figure 1) was implied to be involved in the emitter formation and to account for the differences in the bioluminescence spectra of the hydromedusan photoproteins [5,26]. This assumption is supported by the studies of the photoprotein mutants [27,28,29,30,31], by the crystal structures determined for some mutants with altered emission spectra [28,29,32], and by the recent theoretical studies [33]. The residues of the second His-Trp-Tyr triad are located at hydrogen-bond distances from the 2-hydroperoxy and carbonyl groups of the substrate (Figure 1). Based on the results of studies on various mutants of obelin and aequorin, it was proposed that these residues participate in stabilization of the 2-hydroperoxy group [34] and could be also involved in the formation of an active photoprotein from apoprotein, coelenterazine, and oxygen [35].

The third His-Trp-Tyr triad is located close to the N1 atom of 2-hydroperoxycoelenterazine but additionally includes a water molecule (Figure 1). In accordance with the crystal structures of photoproteins before the bioluminescence reaction [14,22,23,24,25], the OH group of tyrosine resides at hydrogen-bond distances from the N1 atom of the substrate and the water molecule. In turn, the latter can be hydrogen bonded to the nitrogen atom of His (Figure 1). However, after the bioluminescence, the water molecule is found in the place of the OH group of Tyr, i.e., at a hydrogen-bond distance from the N atom of coelenteramide, while Tyr moves away from the substrate-binding cavity to the protein molecule surface [36]. At the same time, the water molecule remains hydrogen bonded with His (Figure 1). This water molecule was proposed to be involved in catalyzing the decarboxylation reaction by the protonation of the dioxetanone anion [26,36] whereas the function of tyrosine consists rather in the stabilization of 2-hydroperoxycoelenterazine and formation of an active photoprotein [35].

Later, the tertiary structures determined for the two conformational states (before and after the bioluminescence reaction) of the obelin mutant with the substitution of Tyr of this triad to Phe showed that there is no water molecule in the cavity before the bioluminescence, but it appears after light emission [39]. Moreover, the water molecule was located at hydrogen-bond distances from the N atoms of both coelenteramide and histidine, i.e., similar to that found for the wild-type Ca^2+^-discharged obelin [36]. The analysis of the spatial structure of the Ca^2+^-discharged obelin Y138F mutant led us to propose that the water molecule enters the inner cavity through the hole on the protein surface [39]. It was also speculated that this water molecule together with the His and Ser residues might be involved in the formation of “proton channel” to transfer proton from solvent to the N atom of the dioxetanone anion (Figure 1).

In order to figure out the possible involvement of these residues and water molecules in proton transfer, we constructed a set of single and double obelin (S47A, H64Q, H64F, W114F, Y138F, S47A/H64Q, S47A/H64F, H64Q/Y138F, H64F/Y138F) and aequorin (S41A, H58Q, H58F, W108F, Y132F, S41A/H58Q, S41A/H58F, H58Q/Y132F, H58F/Y132F) mutants with substitution of amino acids which might participate in “proton channel” formation [39] or affect the proton transfer to residues with different properties of side chains. Further, we investigated the effect of these substitutions on bioluminescent properties as well as determined the tertiary structure of the obelin mutant with the substitution of the key residue (His64 to Phe) of the proposed “proton channel”.

## 2. Results

### 2.1. Bioluminescence Activity and Spectral Properties

Specific activity of bioluminescent protein is a parameter characterizing its capability to transform the chemical reaction energy into light. The obelin and aequorin mutants can be divided into three groups based on the effect of substitutions on specific activity. The first group of mutants with a replacement of Ser with Ala and His with Gln or both amino acids simultaneously either completely retain the bioluminescence activity or lose less than 30% of it (Table 1). The mutants with substitution of His and Tyr to Phe and the double mutants with additional replacement of Ser with Ala preserve 20–60% of the bioluminescence activity and thus constitute another group. The third group comprises mutants with simultaneous replacement of Tyr and His with Phe and with the substitution of Trp to Phe. The appearance of the hydrophobic side chain of Phe instead of the side chains of Tyr and His as well as of the side chain of Trp, which indole N atom is located at hydrogen-bond distance from the water molecule before and after light emission (Figure 1), results in the conversion of photoprotein into pseudo-luciferase, i.e., photoprotein emits light in a Ca^2+^-independent manner. Nonetheless, the addition of calcium increases light emission intensity of these mutants (Table 1).

The maximal light intensity is definitely the most sensitive to mutations among all bioluminescence parameters studied. All single and double substitutions in obelin and aequorin significantly reduce the maximal light intensity; only in the OL_S47A, AV_S41A, AV_H58Q, and AV_S41A/H58Q mutants, the intensities exceed 10% of that of the corresponding wild-type photoprotein (Table 1). Noteworthy is that, although all the replacements decrease the maximal light intensity, the effect of the substitution of His to Phe alone or in combination with another residue on this parameter is always more prominent.

Calcium ions are not strictly required for the bioluminescence of Ca^2+^-regulated photoproteins since, even without them, the photoproteins display a very low level of light emission named the “Ca^2+^-independent luminescence” [40]. Thus, the function of calcium is rather to speed up the rate of the bioluminescence reaction than to trigger the one. The Ca^2+^-independent luminescence is very sensitive to temperature, which significantly enhances its intensity [40], as well as to substitution of amino acids in a photoprotein [41]. Since Ca^2+^-independent luminescence arises owing to decarboxylation of 2-hydroperoxycoelenterazine initiated by the spontaneous motion of the protein molecule, its intensity can serve as an indicator of stability of the photoprotein complex in solution.

The influence of mutations on Ca^2+^-independent luminescence intensity is diverse (Table 1, Figures S1 and S2). The replacement of Trp with Phe (OL_W114F and AV_W108F) and the simultaneous substitutions of Tyr and His to Phe (OL_H64F/Y138F and AV_H58F/Y132F) significantly increase the intensity of Ca^2+^-independent luminescence of obelin and aequorin. Moreover, the intensity declines rapidly over time without reaching the plateau (Figures S1 and S2). There was only one difference—in the case of the AV_H58F/Y132F mutant, the drop of Ca^2+^-independent luminescence was slower than in the case of the corresponding obelin mutant. Conversely, the substitution of His to Phe (OL_H64F and AV_H58F) alone and together with Ser to Ala (OL_S47A/H64F and AV_S41A/H58F) reduces the intensity of Ca^2+^-independent luminescence 30-150-fold depending on the mutant as compared to the corresponding wild-type photoprotein (Table 1). Other mutations also reduce the intensity of Ca^2+^-independent luminescence, but the effect is less pronounced. Noteworthy is that there is no distinct correlation between the influence of mutations on the intensities of Ca^2+^-independent and Ca^2+^-induced light emission.

The effect of mutations on spectral properties is summarized in Table 2. The substitution of Tyr to Phe either alone or jointly with His slightly shifts the absorption maximum toward shorter wavelengths. The replacement of Trp with Phe as well as simultaneous appearance of Phe instead of His and Tyr leads to the emergence of an additional peak at 345 nm corresponding to the reaction product, coelenteramide, in the absorption spectra (Table 2) that is caused by the instability of these mutants evident from their high Ca^2+^-independent luminescence. The unexpected effect of the substitution of Ser on the absorption spectrum has been revealed in the case of aequorin (Table 2). The absorption spectrum of AV_S41A has a maximum at 435 nm with a shoulder at ~470 nm. In the case of a similar mutation in obelin as well as in the double mutants including the replacement of Ser with Ala, these changes in the absorption spectrum are not observed. It is interesting to note that the absorption spectral maximum at 435 nm is characteristic of ctenophore photoproteins, the substrate-binding cavity of which is formed by completely different amino acids as compared to those in hydromedusan photoproteins [42,43].

**Figure 2:**
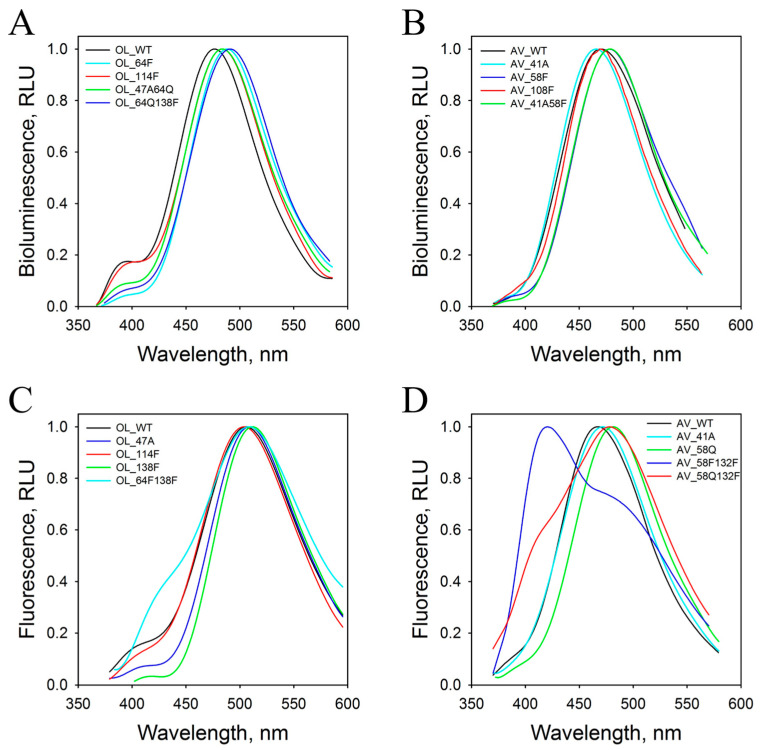
Normalized bioluminescence and fluorescence spectra of obelin (**A**,**C**), aequorin (**B**,**D**) and their mutants.

The wild-type obelin bioluminescence spectrum has a maximum at 480 nm with a shoulder at 400 nm conditioned by light emission from neutral species of coelenteramide [5,44], whereas the wild-type aequorin displays a monomodal spectrum with λ_max_ = 470 nm (Table 2, Figure 2). In the case of obelin, practically all mutations account for a slight red shift (3–12 nm) of the bioluminescence spectrum maximum and reduce the contribution of light emission from neutral species except for the OL_W114F mutant. The spectral characteristics of only the OL_S47A mutant were identical to those of the wild-type obelin.

For aequorin, the influence of amino acid substitutions on bioluminescence is more complicated. Here, practically all mutations result in a slight shift of the spectral maximum to longer wavelengths by 5–15 nm, this being similar to the obelin case, but they also give rise to a small shoulder at 390–395 nm in the emission spectrum (Table 2). In contrast to other aequorin mutants, the spectral maximum of AV_S41A turned out to be shifted by 5 nm toward shorter wavelengths. Interestingly, the homologous mutant of obelin had spectral characteristics similar to those of the wild-type photoprotein. Among all mutants, it was only the unstable AV_W108F mutant that displayed the same spectrum as the wild-type aequorin.

The Ca^2+^-regulated photoproteins with 2-hydroperoxycoelenterazine are non-fluorescent, but they exhibit bright fluorescence at visible wavelengths after the bioluminescence reaction ceases. This is conditioned by the reaction product, coelenteramide, bound within a substrate-binding cavity of the photoprotein. All hydromedusan photoproteins studied can be divided into two groups according to their fluorescence [45]. One group includes Ca^2+^-discharged clytin and obelins from *Obelia longissima* and *Obelia geniculata* with monomodal fluorescence spectra with λ_max_ ranging from 509 to 515 nm, which are red-shifted as compared to their bioluminescence spectra. Another group consisting of Ca^2+^-discharged aequorin and mitrocomin displays blue fluorescence with λ_max_ in the 470–474 nm range. Unlike the fluorescence spectra of the above-mentioned photoproteins, the ones of Ca^2+^-discharged aequorin and mitrocomin coincide with the bioluminescence spectra of these proteins.

By contrast to the bioluminescence spectra, practically all substitutions in obelin lead to the shift of the fluorescence spectral maxima of the Ca^2+^-discharged mutant photoproteins toward shorter wavelengths, which can attain 20 nm (Table 2, Figure 2). There are only two exceptions—the Ca^2+^-discharged OL_H64Q/Y138F mutant, which has the same λ_max_ as the Ca^2+^-discharged wild-type obelin, and the OL_Y138F mutant, which shows a slightly red-shifted fluorescence spectral maximum. In addition, most of the Ca^2+^-discharged obelin mutants display a shoulder in fluorescence spectra at shorter wavelengths with its contribution varying from 5% to 37% depending on the substitutions. Only the mutants with the replacement of His64 to Phe or Gln as well as the OL_S47A/H64F mutant reveal monomodal spectra, i.e., similar to that of the Ca^2+^-discharged wild-type obelin (Table 2).

The effect of mutations on the fluorescence spectrum of the Ca^2+^-discharged aequorin often differs from that on the fluorescence spectrum of the Ca^2+^-discharged obelin, despite the fact that the substrate-binding cavities of these photoproteins are formed by practically the same residues situated in the same positions. Whereas the substitutions in obelin mainly shift the fluorescence spectral maxima toward shorter wavelengths, the same mutations in aequorin either do not influence fluorescence λ_max_ or displace them toward longer wavelengths (Table 2). There was only one exception—the fluorescence spectral maximum of the Ca^2+^-discharged AV_S41A/H58F mutant turned out to be at λ_max_ = 460 nm (Table 2). The most significant changes in the fluorescence spectra are found for the Ca^2+^-discharged aequorin mutants with substitution of Tyr to Phe either alone or together with His. The fluorescence spectra of these Ca^2+^-discharged mutants have a shoulder at shorter wavelengths, which, in effect, becomes the main peak at 420 nm in the case of the AV_H58F/Y132F mutant (Table 2, Figure 2).

### 2.2. Rapid-Mixing Stopped-Flow Kinetics

**Scheme 1:**
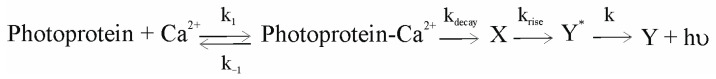
Proposed reaction scheme for photoprotein bioluminescence [47]. X—an intermediate state; Y*—the product, coelenteramide, in the excited state; Y—coelenteramide in the ground state.

Despite the high similarity of the aequorin and obelin structures, the kinetic properties of these photoproteins are significantly different [46]. Whereas the obelin light signal is one of the fastest among the hydromedusan photoproteins, the signal of aequorin is the slowest one. In addition, while the decay kinetics of the aequorin light signal can be satisfactorily characterized by a single rate constant, the decay of the obelin light signal can be well described by a two-exponential function only and consequently by the two rate constants—“fast” (k_decay1_) and “slow” (k_decay2_). The current models describing the bioluminescence kinetics of the Ca^2+^-regulated photoproteins (Scheme 1) attribute the rise (k_rise_) and decay (k_decay_) rates of the light signal to the decarboxylation of the bound 2-hydroperoxycoelenterazine and conformational rearrangements in the photoprotein molecule in response to Ca^2+^ binding, respectively [47,48,49].

**Figure 3:**
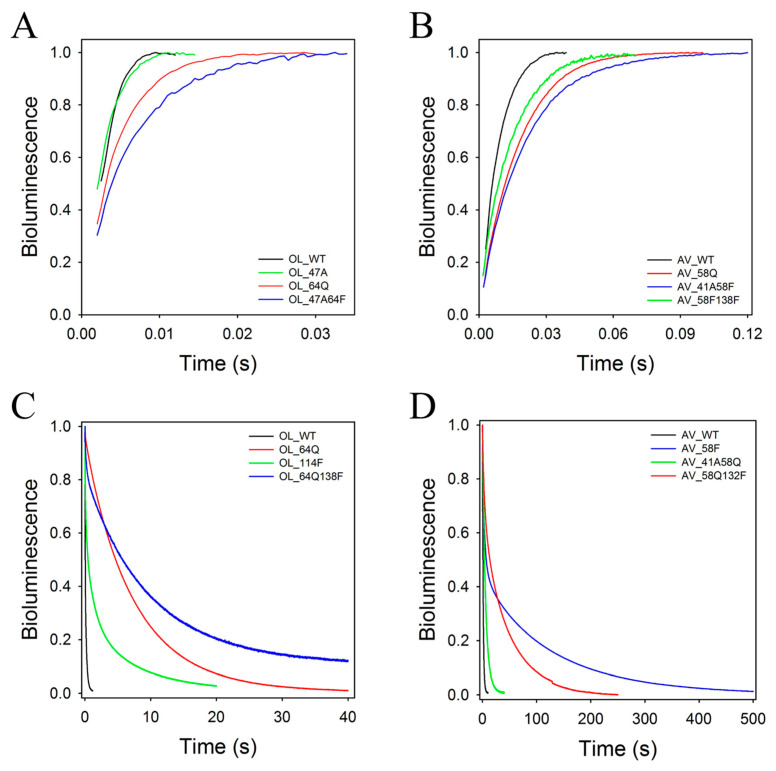
High-speed (**A**,**B**) and low-speed (**C**,**D**) stopped-flow plots of the bioluminescence signals of obelin (**A**,**C**), aequorin (**B**,**D**), and their mutants. The curves are individual shots.

The rate constants of the obelin and aequorin mutants are summarized in Table 3. The substitution of residues in obelin accounts for a decrease in k_rise_ in the case of all mutants, but the most significant decrease in the rise rate of the light signal is observed for mutants with the replacement of His64. For instance, the k_rise_ values of the OL_H64F, OL_S47A/H64F, and OL_H64F/Y138F mutants are 3.5-, 3.2-, and 2.3-fold less, respectively, than that of the wild-type obelin (Table 3, Figure 3). Although the effect of the substitution of His64 to Gln on the rise rate is a little less than in the case of the mutation to Phe, it is also substantial; the k_rise_ for OL_H64Q, OL_S47A/H64Q, and OL_H64Q/Y138F decreases by 1.8–2.4 times as compared to that of the wild-type obelin. The mutations of Ser47 to Ala and Trp114 to Phe only slightly decrease the rates of the rise of the light signals of the corresponding mutants. The substitution of Tyr138 to Phe, which leads to the loss of the water molecule in the substrate-binding cavity [39], causes a 1.8-fold drop in the rise rate of the light signal for the OL_Y138F mutant. It is worth noting that the decrease in the k_rise_ value for this mutant is practically the same as for the OL_H64Q/Y138F mutant (Table 3).

The influence of mutations of the same residues in aequorin on the rate of the light signal rise is more diverse and frequently differs from that revealed for obelin (Table 3). For instance, the most significant drop of k_rise_ (~3.8 times as compared to the wild-type aequorin) is observed in the aequorin mutant with the substitution of Tyr132 to Phe, whereas in the case of obelin it is OL_H64F. Moreover, the rise rate of the light signal in the AV_H58F mutant is practically the same as for the wild-type aequorin. It is worthy of note that the impact of the substitution of His58 to Gln on k_rise_ is higher than that at the replacement of this His with Phe, whereas in the obelin case the effect is the opposite. All the double mutants of aequorin display approximately a 1.7–2.4-fold decrease in the rise rates of the light signals, which matches the range of the AV_H58Q mutant (Table 3). In contrast to obelin, for which all substitutions decrease the k_rise_ values, the AV_S41A and AV_W108F mutants show the increase in the k_rise_ constants (Table 3, Figure 3). The k_rise_ value of the AV_W108F mutant, for example, appeared to exceed almost twice that of the wild-type aequorin.

The effect of mutations on decay kinetics is more complicated. In contrast to the wild-type aequorin, the decay kinetics of the light signals of all its mutants can be satisfactorily described by a two-exponential function only (Table 3). Moreover, the mutations can change the contribution of the “fast” and “slow” components to the decay kinetics. In total, the influence of mutations in obelin on the decay kinetics is very similar to that in aequorin. There is only one distinction—the decay of the light signals of the OL_H64F, OL_S47A/H64Q, and OL_H64F/Y138F mutants with the replaced His can be characterized by a single rate constant, i.e., similar to that as for the wild-type aequorin. However, the values of the decay constants of these mutants are significantly lower as compared to that of the wild-type aequorin; the k_decay_ of the OL_H64F mutant, for instance, is ~230-fold lower. Of note is that the OL_S47A/H64Q mutant having ~7- and a 300-fold lower k_decay_ constant of light signal versus the corresponding constants of the wild-type aequorin and obelin displays the specific bioluminescent activity practically equaling to that of the wild-type photoprotein (Table 1). These properties make this mutant very attractive for analytical applications in vivo and in vitro.

### 2.3. Overall Structure of OL_H64F Mutant

**Figure 4:**
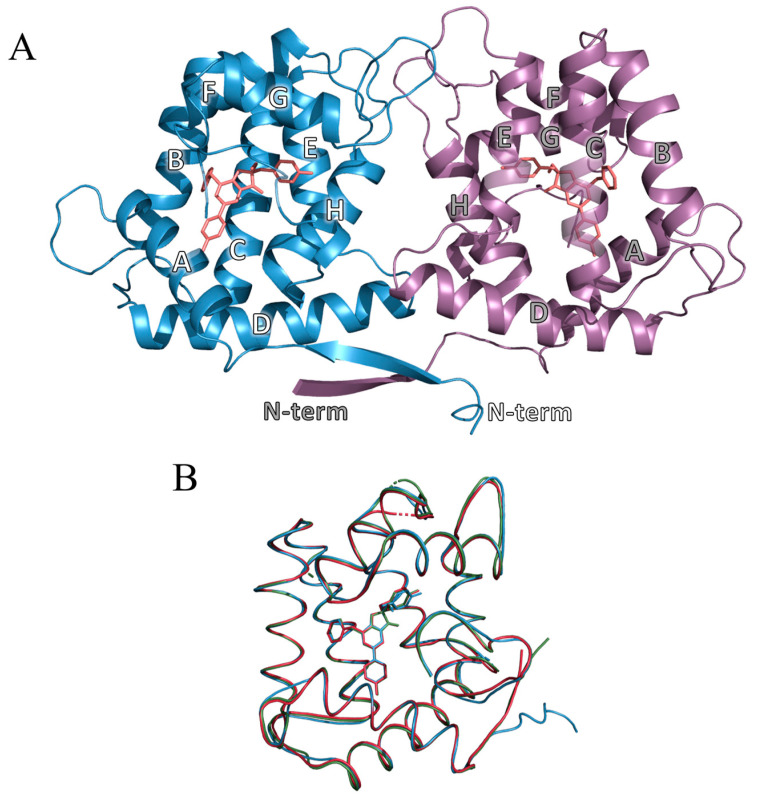
Crystal structure of OL_H64F mutant. (**A**) Overall structure of OL_H64F mutant. The helices are marked by letters A–H. (**B**) Superimposition of OL_H64F mutant (blue, PDB 8C6O, chain A), wild-type obelin (red, PDB 1QV0), and OL_Y138F mutant (green, PDB 4MRX).

The crystal structure of the OL_H64F mutant contains two photoprotein molecules per asymmetric unit (Figure 4A). The final model comprises two protein chains (A, B) consisting of 195 and 194 amino acid residues, correspondingly, two 2-hydroperoxycoelenterazine molecules, and 264 molecules of solvent. The first residue in chain B is not visible in the electron-density maps. The RMSD of the Cα atoms of chain A vs. chain B is only 0.23 Å, which definitely shows a high structural identity of chains A and B. Minor differences were found in orientations of some side chains on the surface molecules, labile regions of loops, and *N*-termini. The most significant distinction was revealed in the loops formed by the residues 119–129, which are the part of the Ca^2+^-binding site III, this being apparently due to the contacts between the two dimer chains as well as the intramolecular interaction between Asp125 and Ser127 in chain B and the lack of it in chain A.

The OL_H64F mutant, similar to the wild-type photoproteins and their various mutants, is a globular molecule with the radius of ~25 Å. It is formed by the two sets of four helices designated as A–D and E–H in the *N*- and *C*-terminal domains, respectively (Figure 4A). The overall structure of the OL_H64F mutant strongly matches those of the wild-type obelin (PDB: 1QV0) [24] and the OL_Y138F mutant (PDB: 4MRX) [39] bound with the 2-hydroperoxy adduct of coelenterazine—the RMSD of the Cα atoms of the OL_H64F chain A vs. wild-type obelin (PDB: 1QV0) and the OL_Y138F mutant (PDB: 4MRX) is 0.37 Å and 0.28 Å, respectively (Figure 4B).

The electron densities detected at the Ca^2+^-binding loop I in both protein chains A and B (residues 29–37) are conditioned by the bound Na^+^, just as it was found in the wild-type obelin [50] and Ca^2+^-discharged obelin-*v* [51]. The ion is octahedrally coordinated by Asp30, Asn32, Asn34, Lys36, and two or one water molecules in the chain A and B, respectively. Although the coordination geometry is typical for both calcium and sodium ions, no calcium was added to the crystallization solution and hence the ion was recognized as sodium.

In the Ca^2+^-regulated photoproteins, the *C*-terminus caps the substrate-binding cavity owing to the hydrogen-bond interactions between the residues situated in helixes A and H as well as in the *C*-terminal sequence [22,23,24,25]. This provides the inaccessibility of the internal substrate-binding cavity of the photoproteins for solvent and, consequently, optimizes the efficient population of the first electronic excited state of coelenteramide favoring a high quantum yield of its fluorescence. In the OL_H64F mutant, the hydrogen-bond network ensuring isolation of the inner cavity from solvent exactly corresponds to that in the wild-type photoproteins [14,52]—the Nε_2_ atoms of His22 and His24 (helix A) are hydrogen bonded with carbonyl oxygens of Trp179 (helix H) and Gly193 (*C*-terminus), Nη_1_ and Nη_2_ atoms of Arg21 (helix A)—with carbonyl oxygen of Phe178 (helix H), Oδ1 atom of Asp187 (*C*-terminus), and oxygen of *C*-terminal Pro195.

Thus, the comparison of the OL_H64F mutant structure with those of the wild-type obelin and the OL_Y138F mutant certainly evidences that the substitution of His to Phe does not change the overall structure of this mutant.

### 2.4. Structure of the Substrate-Binding Cavity of OL_H64F Mutant

The residues surrounding the 2-hydroperoxycoelenterazine molecule within the inner cavity of the OL_H64F mutant at the distance of 4 Å from its atoms are practically identical to those in the wild-type obelin (PDB code 1QV0) and are allocated in all the helices forming the photoprotein molecule (Figure 2A): A (His22, Met25 and Leu29), B (Ile42 and Ile50), C (Phe72), D (Phe88 and Trp92), E (Ile111, Trp114, Gly115, Val118 and Phe119), F (Trp135 and Phe138), and H (Met171, His175, and Trp179). In addition, Ile144 and Tyr190 originate from the loop linking helices F and G and the protein C-terminus, respectively. There were only two differences found—Val118 located in the helix E appeared within the distance of 4 Å while Ile45 of the helix B was shifted beyond it.

**Figure 5:**
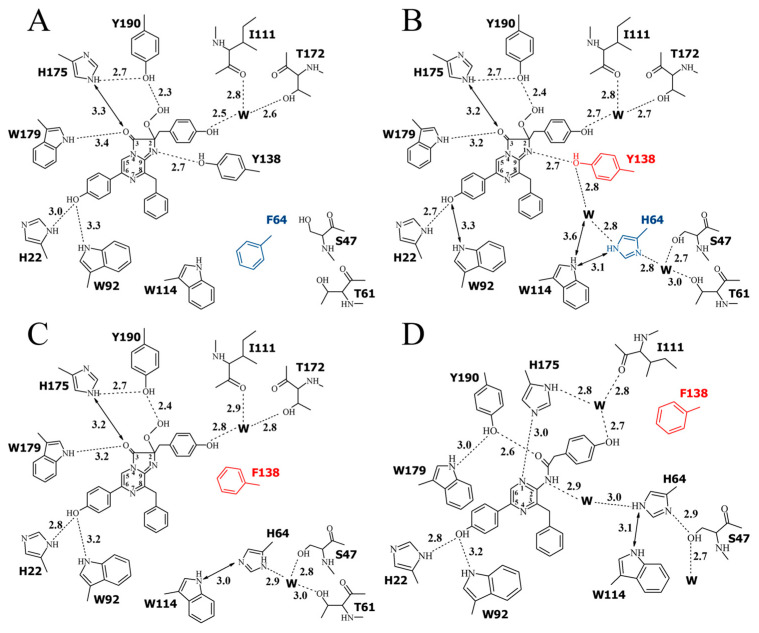
Two-dimensional representation of the hydrogen bond network. (**A**) OL_H64F mutant (PDB 8C6O, chain A), (**B**) wild-type obelin (PDB 1QV0), (**C**) active OL_Y138F mutant (PDB: 4MRX), and (**D**) Ca^2+^-discharged OL_Y138F mutant (PDB 4MRY). Mutated residues H64(F64) and Y138(F138) are shown in blue and red, respectively. Hydrogen bonds are shown as dashed lines, distances between atoms are shown as arrows, and “W” stands for a water molecule. Distances are given in Å.

The hydrogen-bond network formed by the side chains of the key residues involved in the substrate decarboxylation and excited state formation [5] and by the 2-hydroperoxycoelenterazine atoms in the OL_H64F obelin mutant is shown in Figure 5. For comparison, the hydrogen-bond networks formed by the same residues in the wild-type obelin [24] and the active OL_Y138F and the Ca^2+^-discharged OL_Y138F mutant [39] are also presented. In the active photoproteins, the hydrogen bonds formed by His22 and Trp92 with the oxygen atom of the OH group of the 6-(*p*-hydroxyphenyl) substituent of 2-hydroperoxycoelenterazine, Tyr190 with the hydroperoxide group, His175 with Tyr190 and C3 carbonyl oxygen of the substrate are present in the wild-type obelin and in both its mutants, and the hydrogen-bond distances are practically identical in these proteins (Figure 5).

**Figure 6:**
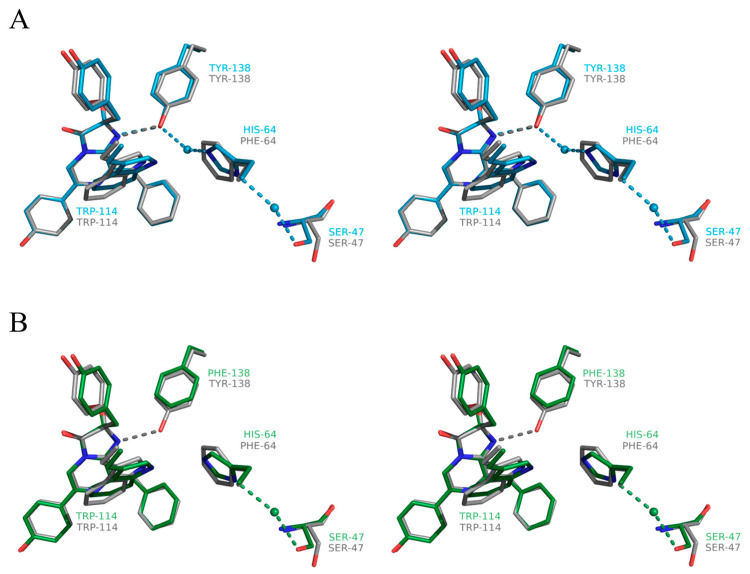
Stereoview of the superimposition of 2-hydroperoxycoelenterazine molecules with the residues residing near their N1 atoms: (**A**) OL_H64F mutant (gray) vs. wild-type obelin (cyan) and (**B**) OL_H64F mutant (gray) vs. OL_Y138F mutant (green). Hydrogen bonds are shown as dashed lines. Oxygen and nitrogen atoms are colored with red and blue, respectively.

As expected, similar to the OL_Y138F mutant, the main changes in the substrate-binding cavity of the OL_H64F mutant occur near the N1 atom of the 2-hydroperoxy adduct of coelenterazine. In the wild-type obelin, the OH group of Tyr138 is hydrogen bonded with the N1 atom of 2-hydroperoxycoelenterazine and the water molecule, and His64 is at hydrogen-bond distances with the water molecules. The appearance of the hydrophobic side chain of Phe instead of the hydrophilic chain of His hinders the access of the water molecules in this part of the substrate-binding cavity but does not influence the hydrogen bond between Tyr138 and the N1 atom of the substrate (Figure 5). It should be noted that Phe is found in the same position as His in the wild-type obelin and the OL_Y138F mutant (Figure 6), i.e., the side chain of Phe does not drastically disturb the structure of this part of the inner cavity.

The crystal structures of the Ca^2+^-regulated photoproteins after the bioluminescence reaction, i.e., bound with a product, coelenteramide, and calcium ions, are available for the wild-type obelin [36] and the OL_Y138F mutant [39]. According to these structures, both Tyr138 in the Ca^2+^-discharged wild-type obelin and Phe138 in the Ca^2+^-discharged OL_Y138F mutant go away from the substrate-binding cavity, while His is slightly moved toward the N atom of coelenteramide. In both structures, the side chain of His appeared to be hydrogen bonded with the N atom of coelenteramide through a water molecule “bridge”. As the water molecule was absent in the internal cavity of the active OL_Y138F mutant, it was speculated that the one might penetrate into the cavity through a hole on the protein molecule surface [39]. Even though the attempts to crystallize the Ca^2+^-discharged OL_H64F mutant were not successful, we propose that the Phe residue might reside in the same position as before the bioluminescence. This assumption looks plausible taking into account that the His residue does not drastically change its position after the bioluminescence reaction in both the Ca^2+^-discharged wild-type obelin and the Ca^2+^-discharged OL_Y138F mutant. It means that the water molecule will be also missing in the cavity of the Ca^2+^-discharged OL_H64F mutant owing to the presence of the hydrophobic side chain of Phe.

## 3. Discussion

Although numerous experimental and theoretical studies on the bioluminescence of the Ca^2+^-regulated photoproteins have been carried out, many features of the functioning of these unique bioluminescent proteins are still unclear. Moreover, the conclusions made from theoretical calculations are often not in agreement with those derived from the experimental results. The main discrepancies concern the ionic form of the dioxetanone intermediate from which its decomposition to yield a product, coelenteramide, in excited state, occurs, and also the role of the water molecule situated in a proximity to the N1 atom of 2-hydroperoxycoelenterazine in this process.

Based on the results of studies on the chemiluminescence of coelenterazine analogues in aprotic solutions, McCapra and Chang [53] proposed a mechanism for the oxidation of coelenterazine and the formation of a product in an excited state. Oxidative decarboxylation of coelenterazine proceeds through the formation of several intermediates. The reaction of coelenterazine with oxygen produces a primary oxygenated product, C2-hydroperoxy anion, which is rapidly converted to the dioxetanone anion. Its decomposition then leads to the formation of the amide anion of coelenteramide in an excited state. The reaction mechanism is now generally accepted to be the same for all bioluminescent proteins utilizing coelenterazine as a substrate. Later, based on the similarity of the emission spectra of aequorin and the fluorescence spectra of the amide anion of coelenteramide [54] this amide anion in the excited state was presumed to be the emitter in the aequorin light-emitting reaction.

Determination of the crystal structures of aequorin and obelin with 2-hydroperoxycelenterazine [22,23,24] and obelin with the reaction product, coelenteramide [36,50] allowed identification of the amino acid residues of the substrate-binding cavity of the photoproteins before and after the bioluminescence. On the strength of these findings and the results of studies of the chemiluminescence of coelenterazine analogues [55] and the fluorescence of coelenteramide in various solvents [44], the “proton-relay” mechanism was proposed [5,26]. It suggests the functional role of several residues of the substrate-binding cavity of photoprotein in the decarboxylation reaction and emitter formation. The water molecule, being in a proximity to the N1 atom of 2-hydroperoxycelenerazine, forming the hydrogen bonds with the Tyr138 and His64 residues and being at the hydrogen-bond distance from the N atom of coelenteramide after the bioluminescence, was inferred to catalyze the decarboxylation by the protonation of the dioxetanone anion prior to its decomposition. As a result, neutral dioxetanone is formed, and its subsequent decomposition leads to the formation of a primary emitter, neutral coelenteramide, which emits light at λ_max_ = 390–400 nm [44]. The excited state of coelenteramide emitting light at longer wavelengths is believed to be the excited phenolate anion arising as a result of proton dissociation of the OH group of the 6-(*p*-hydroxyphenyl) substituent of coelenterazine in the direction to His22, which is located within the hydrogen-bond distance (Figure 5). The phenolic group p*K* in excited state is several units below its ground state value [56], and if it falls below 6.5 (the expected p*K* of His) rapid transient proton dissociation and its “transient displacement” toward the N atom of His will take place. This process is synchronously accompanied by the generation of the phenolate anion in the excited state. Since its fluorescence life-time is 5–6 ns [57], there is enough time for “proton displacement” to occur before light emission.

The proposed “proton-relay” mechanism accounts for the light emission spectra of the hydromedusan photoproteins and is in a good agreement with numerous previous results on the chemiluminescence of the coelenterazine analogues and coelenteramide fluorescence. The spatial structures of the Y138F [39] and F88Y [32] obelin mutants in two conformational states also counts in favor of the proposed mechanism. This hypothesis is also based on the research conducted by Usami and Isobe [58] on the chemiluminescence of a coelenterazine model compound. They detected a chemiluminescence emission after the photooxygenation of the model compound at low temperature. The resulting product was trapped, and its structure was determined as a dioxetanone derivative by low-temperature NMR. On warming, the product primarily emitted light at shorter wavelengths (λ_max_ = 400 nm) and then at longer wavelengths (λ_max_ = 475 nm) that corresponded to the emission from neutral and anion forms of coelenteramide in the excited state, respectively. Relying on this finding, it was inferred that the dioxetanone anion is more stable as compared to the neutral dioxetanone intermediate. Moreover, it was proposed that there are two routes for decarboxylation of dioxetanone derivative—one from the neutral dioxetanone and another from the dioxetanone anion, and that the conversion of the excited anion coelenteramide to the excited neutral form cannot occur under the low temperature even at acidic conditions [58]. It means that the decomposition of the neutral dioxetanone formed in the inner cavity of the photoproteins can yield a product in an excited state.

**Figure 7:**
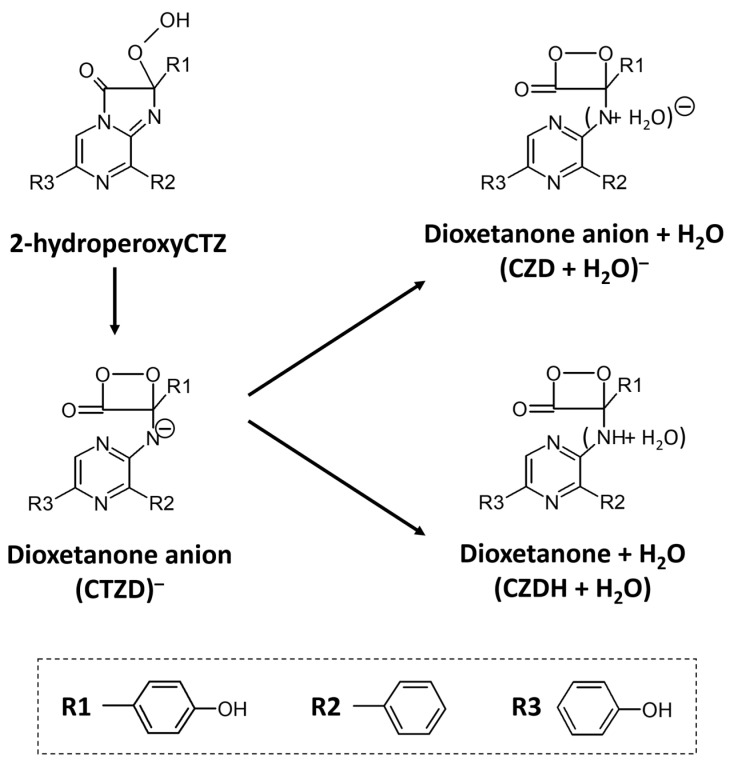
Dioxetanone derivatives that might be formed in bioluminescent reaction of hydromedusan photoproteins.

In recent theoretical studies, another mechanism of decomposition of dioxetanone was proposed [59]. The author considered three QM models for decomposition of coelenterazine dioxetanone that took into account the water molecule and different dioxetanone protonation states—(CZD + H_2_O)^−^, (CZDH + OH)^−^, and (CZDH + H_2_O) (Figure 7). It was concluded that decomposition of the dioxetanone anion in the (CZD + H_2_O)^−^ model goes through a charge-transfer (CT) catalyzed asynchronous-concerted process, which can be elucidated by the gradual reversible CT initiated luminescence (GRCTIL) mechanism, and which provides the highest quantum yield of singlet chemiexcitation. The decomposition of neutral dioxetanone in the (CZDH + H_2_O) model occurs via an uncatalyzed non-CT biradical process that can lead to the production of a large amount of non-fluorescence triplet product [59]. The suggested mechanism was consistent with those of firefly squid [60] and firefly [61]. The presence of a shoulder at shorter wavelengths in the bioluminescence spectrum of obelin corresponding to the emission from the excited neutral coelenteramide was assigned to the fast protonation of the excited coelenteramide anion before its relaxation to the ground state [59]. Recently, it was confirmed by molecular dynamic (MD) simulations and the hybrid quantum mechanics/molecular mechanics (QM/MM) method [62]. According to the calculations, a low activation barrier as well as strong hydrogen-bond network between the proton donor (histidine) and the proton acceptor (anionic S_1_-coelenteramide) with a water molecule as a bridge promotes fast proton transfer to the excited anionic S_1_-coelenteramide before its transition to the ground state.

The relevant experimental and theoretical studies on the chemiluminescence of imidazopyrazinone-type substrates were performed not long ago [63,64]. The thermolysis of both amide and neutral dioxetanones proceeds by a stepwise biradical mechanism. However, while in the case of the amide dioxetanone the biradical formation occurs due to an electron transfer between moieties, for neutral dioxetanone the biradical originates on account of the homolytic cleavage of the peroxide bond. This distinction dictated different activation energies for amide (11.5 kcal mol^−1^) and neutral (23.2 kcal mol^−1^) species. However, the activation barrier for the neutral species turned out to correspond well to those measured experimentally for other dioxetanones and dioxetanes (~20 kcal mol^−1^) [65,66,67,68]. Thus, even though the activation barrier for neutral dioxetanone exceeded that of amide dioxetanone, it was proposed that the neutral species cannot be ruled out from being responsible for efficient chemiexcitation, based on energetic criteria only [64]. Moreover, according to the computations, the singlet chemiexcitation for the neutral dioxetanone was significantly more efficient owing to the presence of a flat and long region of the potential energy surface found in the thermolysis of neutral dioxetanone, in which both S_0_ and S_1_ are nearly degenerated. In addition, the analysis carried out for the potential effect of amino acids that might be involved in the formation of active sites of the enzymes using imidazopyrazinone-type substrates on the neutral-amide chemical equilibrium showed that cationic amino acids can easily protonate the amide dioxetanone into the neutral species. Reasoning from these findings, it was proposed that even if the amide dioxetanone is the primary decomposition product of the bioluminescence reaction, the one might easily be converted into a neutral dioxetanone within the active site of the luciferase or photoprotein with the involvement of the cationic amino acids such as His or Lys [64]. The results of further experimental and theoretical studies on the chemiluminescence of the imidazopyrazinone-type substrates performed in different aprotic solvents at different pH provided additional support to the proposed mechanism [69]. It turned out that the chemiluminescence yield is higher at acidic pH, whereas the increase in the solution pH results in the inhibition of the chemiluminescence. This phenomenon was supposed to be responsible for the deprotonation of the neutral dioxetanone prior its chemiexcitation [69].

Substitutions of amino acid residues presumably involved in the proton transfer pathway [39] mostly affect the maximal light intensity, specific bioluminescence activity, and stability of the photoprotein complex in the case of the appearance of two Phe residues instead of Tyr and His (Table 1 and Table 3), but they practically have no effect on the spectral characteristics of light emission (Table 2). For instance, the replacement of histidine, the key residue of the “proton channel”, with Phe significantly reduces both the maximal light intensity and the level of the Ca^2+^-independent luminescence of obelin and aequorin. However, the decrease in their specific activities is ~70% of those of the corresponding wild-type photoproteins. Despite the fact that the maximal light intensities are substantially reduced as a result of the mutation of His to Phe, the rates of the rise of the light signals are less sensitive to this replacement. Whereas the k_rise_ value for the OL_H64F mutant light signal is 3.5-fold lower as compared to that of the wild-type obelin, the k_rise_ constant of the corresponding aequorin mutant is practically the same as for the wild-type photoprotein. These effects of His substitution can stem from the absence of the water molecule in the substrate-binding cavity near the N1 atom of 2-hydroperoxycoelenterazine (Figure 5).

Although the attempts to crystallize the OL_H64F mutant in conformation after the bioluminescence were not successful, we can reasonably suggest that the water molecule is also missing in this location after the reaction. In the case of the Y138F mutant or wild-type obelin in conformations after the bioluminescence reaction, either Tyr or Phe moved away from the inter cavity [36,39] providing the space for the water molecule near the N atom of coelenteramide. This water molecule may enter either through the hole on the photoprotein surface in the case of the Y138F mutant [39] or may be shifted from its previous position before the reaction in the case of the wild-type obelin [36]. In both cases, the His residue, being shifted toward the N atom of coelenteramide, remains within the substrate-binding cavity. We can plausibly assume that in the case of the OL_H64F mutant and most likely in the case of the similar aequorin mutant, Phe might also stay within the inner cavity as does His, and that its hydrophobic side chain might consequently hinder the appearance of the water molecule near the dioxetanone anion N atom. Thus, the ionic form of the dioxetanone anion (CTZD^−^) is only produced during the reaction and, hence, the amide anion in an excited state is only formed as a result of the CTZD^−^ decomposition in the case of the replacement of His to Phe (Figure 7). However, it causes the reduction in specific activity and maximal light intensity (Table 1 and Table 3). These effects definitely show that the water molecule is strictly required for effective bioluminescence and, therefore, the decomposition of dioxetanone occurs from either its anion (CZD + H_2_O)^−^ or neutral (CZDH + H_2_O) form (Figure 7).

In contrast, although the light intensity and specific activity also decrease with the appearance of Gln having hydrophilic uncharged side chain instead of His, the reduction is much less against those in the case of the obelin and aequorin mutants with a replacement of His with Phe (Table 1 and Table 3). This might take place through hydrogen bonding of the Gln amine group, even though it is uncharged, with the water molecule [70]. Although it is obvious that this hydrogen bond is weaker than that formed by His, nevertheless Gln can provide positioning of the water molecule near the N atom of the dioxetanone anion. However, Gln cannot ensure the proton transfer to the dioxetanone anion in contrast to His and, consequently, only the formation of (CZD + H_2_O)^−^ is possible. Noteworthy is that the effect of the simultaneous substitution of His to Gln and Tyr to Phe on light intensities and the specific activities of both photoproteins is practically identical to that in the case of the replacement of His to Phe only (Table 1 and Table 3). This may be due to the absence of the water molecule near the N atom of the dioxetanone anion on account of the uncharged side chain of Gln which, unlike the His side chain, cannot attract a water molecule capable of permeating through the surface hole, as was suggested based on the crystal structure of the Ca^2+^-discharged Y138F obelin mutant [39]. Thus, only the ionic form of CTZD^−^ can be formed.

The replacement of Ser in obelin and aequorin, which is the first residue of the “proton channel” connecting the inner cavity of the photoprotein with a solvent to Ala reduces the maximal light intensity several times but only slightly affects the specific activity and the k_rise_ value as compared to the corresponding wild-type photoprotein (Table 1 and Table 3). The appearance of Ala instead of Ser in this position may hamper the transfer of the proton from solvent towards the dioxetanone anion, which, in turn, will facilitate the formation of (CZD + H_2_O)^−^. At the same time, we cannot exclude that some other residue (Thr61, for example (Figure 5)) may take the function of Ser providing the formation of the neutral form of dioxetanone but possibly with less efficiency.

The studies of the bioluminescent properties of the obelin and aequorin mutants as well as the crystal structures of the OL_H64F and Y138F [39] obelin mutants show that, depending on the presence or absence of the water molecule near the N atom of the 2-hydroperoxy adduct of coelenterazine, three different dioxetanone species could be formed—CTZD^−^, (CZD + H_2_O)^−^, and (CZDH + H_2_O) (Figure 7). However, based on the bioluminescent properties of the obelin and aequorin mutants, the decomposition of only the neutral form of dioxetanone (CZDH + H_2_O) yields the highest specific activity and the maximal rate of the bioluminescence reaction. This clearly indicates that, as it was proposed earlier [5], the water molecule yet performs the “catalytic function” through the protonation of the dioxetanone anion prior to its decomposition but does not serve as a simple “bridge” for the proton transfer to the coelenteramide anion in the excited state as it was suggested in [62]. This conclusion contradicts the one derived from theoretical studies [59], according to which the decomposition of (CZD + H_2_O)^−^ provides the highest quantum yield of singlet chemiexcitation. However, it is strongly supported by other theoretical and experimental data showing that decomposition of the neutral dioxetanone cannot be ruled out from being responsible for efficient chemiexcitation considering energetic criteria only [63,64,69].

## 4. Materials and Methods

### 4.1. Materials

Coelenterazine was obtained from NanoLight Technology, a division of Prolume Ltd. (Pinetop, AZ, USA). Other chemicals, unless otherwise stated, were from Sigma-Aldrich (St. Louis, MO, USA) and the purest grade available.

### 4.2. Site-Directed Mutagenesis and Preparation of the Photoprotein Samples

Obelin and aequorin mutants were obtained by site-directed mutagenesis on the pET19-OL8 and pET22-A7 plasmids for *Escherichia coli* expression carrying the *O. longissima* apo-obelin gene [37] and the *A. victoria* apo-aequorin gene with its *N*-terminus truncated by six amino acid residues [38], respectively. Site-directed mutagenesis was carried out using the QuikChange site-directed mutagenesis kit (Agilent, La Jolla, CA, USA) according to the protocol supplied with the kit. The plasmids harboring mutations were verified by DNA sequencing (SB RAS Genomics Core Facility, Novosibirsk, Russia).

For apophotoprotein production, the transformed *E. coli* BL21-Gold (DE3) Codon Plus (RIPL) cells were cultivated with vigorous shaking at 37 °C in LB medium containing ampicillin (200 µg/mL). The induction was initiated with 1 mM IPTG when the culture reached an OD_590_ of 0.6–0.8. After addition of IPTG, the cultivation of cells was continued for another 3 h. Obelin and aequorin as well as their mutants were purified and activated with coelenterazine, as described elsewhere [45,71,72]. Coelenterazine concentration in the methanol stock solution was determined spectrophotometrically using the extinction coefficient ε_435nm_ = 9800 M^−1^cm^−1^ [1]. Active photoproteins were separated from apophotoproteins and coelenterazine excess by chromatography on HiTrap Q HP column (GE Healthcare). The freshly purified obelin, aequorin, and their mutants were immediately used in the experiments.

Protein concentration of active photoproteins was determined spectrophotometrically using the extinction coefficient A_1%,1cm_ at 460 nm equal to 1.06 [45,73].

### 4.3. Crystallization, Data Collection, Structure Solution, and Crystallographic Refinement

For crystallization, the OL_H64F mutant obtained after ion-exchange chromatography was exchanged into a buffer consisting of 2.5 mM EDTA, 20 mM Tris-HCl pH 7.2 and was concentrated to ~10 mg/mL using Amicon Ultra Centrifugal Filters (10 kDa) (Merck Millipore, Burlington, MA, USA).

Protein crystals were obtained by the sitting-drop vapor-diffusion method in 96-well crystallization plates (SPT Labtech, Melbourn, UK). For screening initial crystallization conditions, the NT8 crystallization robot (Formulatrix, Bedford, MA, USA) and commercially available crystallization screening kits JCSG, PACT, and SG1 (Molecular Dimensions, Sheffield, UK) were used. Each sitting drop contained 200 nL of protein solution and 200 nL of reservoir solution. All hits were optimized manually using hanging-drop vapor-diffusion technique. The best condition for crystallization of H64F obelin was a solution of 2.1 M malic acid pH 7.0 in the drop containing 1 µL of protein solution and 1µL of reservoir solution. Thereafter, the OL_H64F mutant crystals were grown for 3–7 days at 20 °C to the final size of 100 μm. For X-ray diffraction analysis, the crystals were harvested from the crystallization drop using nylon loops and were flash-cooled in liquid nitrogen. Prior to freezing, the crystals were cryoprotected by soaking in crystallization solution containing 25% vol/vol glycerol for several seconds.

The data from the H64F obelin crystals were collected on beamline ID30A-3 using Eiger_4M detector at the European Synchrotron Radiation Facility (ESRF), France [74]. Native diffraction data were indexed, integrated, and scaled in P6_1_ space group using the XDS software (v.20220120) [75]. The initial model was obtained by molecular replacement (MR) pipeline of Autorickshaw [76] using the AlphaFold structure of the wild-type obelin AF-Q27709-F1 as a search model. After that, the model was iteratively refined with PHENIX [77] and adjusted manually using Coot [78]. Visualization and superimposition of the molecular structures were performed using PyMOL 2.5.0 (Schrödinger, LLC, New York, NY, USA). The parameters to detect hydrogen bonds were 3.6 Å for an ideal geometry and 3.2 Å for minimally acceptable geometry, 180° for a hydrogen-bond cone, and 63° for the maximal hydrogen-bond angle [79]. The RMSD was calculated using Align method of PyMOL 2.5.0. The structure of H64F obelin was determined with a final resolution of 2.2 Å and deposited at PDB bank under ID 8C6O (Table 4).

### 4.4. Bioluminescence Assay

Bioluminescence was measured by a luminometer BLM-003 (Oberon-K, Krasnoyarsk, Russia) equipped with a photon-counting head H10682-01 as a light detector (Hamamatsu Photonics, Hamamatsu, Japan) by rapid injection of 10 µL aliquot of a solution (~0.35 M NaCl, 5 mM EDTA, 20 mM Tris-HCl pH 7.2) containing the photoprotein with a constant-rate syringe CR 700-20 (Hamilton, Reno, NV, USA) into a luminometer cell containing 490 µL of 2 mM CaCl_2_ in 50 mM Tris-HCl pH 8.5 at 23 °C. The temperature of the assay tube was supported with a temperature Peltier-controlled cell holder. The luminometer was supplied with a set of neutral filters to extend the linear detection range. The bioluminescence signal was recorded until the bioluminescent reaction ceased and then integrated to calculate the specific activity. The specific activities were calculated by averaging five shots for each photoprotein sample.

Ca^2+^-independent luminescence [40] was determined by adding 500 µL of photoprotein solution in 5 mM EDTA, 20 mM Tris-HCl pH 7.2 into a luminometer cell at 23 °C. The intensity of Ca^2+^-independent luminescence activity was measured when bioluminescent signal remained at the same level for at least 10 min.

The recombinant Ca^2+^-regulated photoprotein aequorin as a light standard [73] was employed to calibrate the luminometer in photons.

### 4.5. Spectral Measurements

The absorption spectra were obtained with an UV-2600 double-beam spectrophotometer (Shimadzu, Kyoto, Japan). The bioluminescence and fluorescence spectra were measured using a Cary Eclipse spectrofluorometer (Agilent Technologies, Santa Clara, CA, USA). The slit width was 5 nm. The bioluminescence spectra were measured in 50 mM Bis-Tris propane-HCl pH 7.0. Bioluminescence was initiated by injection of CaCl_2_ solution into 100 mM Tris-HCl pH 7.2. The concentration of free calcium was ~0.5 mM in order to provide an approximately constant light level during the spectral scan. In the case of a substantial change in the bioluminescence intensity, the data points were also corrected for the bioluminescence decay. The fluorescence spectra of Ca^2+^-discharged photoproteins were recorded after the bioluminescence reaction ceased. The bioluminescence and fluorescence spectra were corrected for the detector spectral sensitivity with an algorithm supplied with the instrument.

### 4.6. Rapid-Mixing Kinetics Measurements

Kinetic measurements were performed with EDTA-free solutions. EDTA was removed from the purified proteins by gel filtration on a 1.5 × 6.5 cm D-Salt dextran desalting column (Pierce). The column was equilibrated, and the protein was eluted with 150 mM KCl, 5 mM PIPES (piperazine-1,4-bis(2-ethanesulfonic acid)), pH 7.0 previously passed (twice) through freshly washed beds of Chelex 100 chelating resin (Sigma-Aldrich) to remove the trace amounts of Ca^2+^. To exclude possible contamination with EDTA, only the first few protein fractions to come off the column were used for rapid-mixing measurements.

The light response kinetics after sudden exposure to a saturating Ca^2+^ concentration was examined with an SX20 stopped-flow machine (cell volume 20 µL, dead-time 1.1 ms) (Applied Photophysics, Leatherhead, UK). The temperature was controlled with a circulating water bath and was set at 20 °C in all experiments. One syringe contained 40 mM CaCl_2_, 30 mM KCl, 5 mM PIPES buffer, pH 7.0; another one—Ca^2+^-free photoprotein solution of the same ionic strength: 150 mM KCl, 5 mM PIPES, pH 7.0 [46]. Before measurements, both syringes were prewashed with the EGTA solution and then thoroughly with deionized water. The solutions were mixed in equal volumes; thus, the final Ca^2+^ concentration in the reaction mixture was 20 mM.

The rise rate constant was calculated by one-exponential fit with Sigma Plot 11 software as described elsewhere [46]. The decay rate constants were calculated by one or two-exponential fitting. The contribution of decay rate constants k_decay1_ and k_decay2_ was estimated as the relative amplitude calculated from the fitted amplitudes a and b with their sum normalized to 1 [34]. All constants were calculated using averaging of five independent shots.

## 5. Conclusions

In the present work, using a site-directed mutagenesis approach, we have addressed the role of amino acid residues of obelin and aequorin which, as previously suggested [39], may be involved in the proton transfer from solvent to the dioxetanone anion. We demonstrate that despite some variations, in general the substitution of these residues leads to similar effects on the bioluminescence characteristics of obelin and aequorin—the maximal light intensity, the rise rate of the light signal, and the intensity of the Ca^2+^-independent luminescence are decreased, but the bioluminescence spectrum is not significantly affected. To elucidate the structural basis of the influence of mutations on the bioluminescent properties of the photoproteins, we have also determined the crystal structure of the OL_H64F mutant in conformation before the bioluminescence reaction. The substrate-binding cavity of this mutant turned out to contain no water molecule, i.e., as it was found for the OL_Y138F mutant [39]. However, in contrast to the OL_Y138F mutant, this water molecule could hardly appear after the bioluminescence reaction on account of the presence of hydrophobic side chain of Phe.

Thus, on the ground of the bioluminescent properties of the obelin and aequorin mutants as well as spatial structures of the obelin mutants, the conclusion has been made that the water molecule is strictly required for effective bioluminescence and, consequently, that it rather performs the “catalytic function” [5] than serves as a “bridge” for the proton transfer to protonate the excited anionic S_1_-coelenteramide before its relaxation to the ground state [62]. Moreover, based on our findings, the functional role of each amino acid residue forming the Tyr-His-Trp triad near the N1-atom of the 2-hydroperoxy adduct of coelenterazine might be attributed. It is most likely that Trp in this position facilitates the proper positioning of the 2-hydroperoxycoelenterazine molecule within the inner cavity as its substitution destabilizes the photoprotein molecule. Tyr is apparently involved in the formation of an active photoprotein complex, as was proposed earlier [35], and probably in positioning the water molecule before the reaction. The key residue of the Tyr-His-Trp triad is His since, in fact, along with the water molecule, it performs the “catalytic function” by transferring the proton from solvent to the dioxetanone anion to generate its neutral ionic state (CZDH + H_2_O). According to our results, only decomposition of this form of dioxetanone can provide the highest light output in the light-emitting reaction of the hydromedusan photoproteins.

## Figures and Tables

**Table 1:** Bioluminescence properties of obelin and aequorin mutants.

Photoprotein	Maximal Light Intensity (Quanta/mg) ^a^, ×10^13^	Specific Activity (Quanta/mg) ^a^, ×10^15^	Relative Specific Activity	Ca^2+^-Independent Luminescence (Quanta/mg) ^a^, ×10^3^
Obelin				
OL_wt	62.19 ± 2.00 (1) ^b^	6.72 ± 0.04	1	70.80 ± 0.88 (1) ^c^
OL_S47A	9.34 ± 0.74 (0.15)	6.44 ± 0.05	0.96	21.91 ± 0.31 (0.31)
OL_H64F	0.32 ± 0.004 (0.05)	2.41 ± 0.06	0.36	1.13 ± 0.12 (0.016)
OL_H64Q	1.44 ± 0.08 (0.02)	5.40 ± 0.11	0.80	17.10 ± 0.30 (0.24)
OL_W114F	4.91 ± 0.71 (0.08)	0.94 ± 0.01	0.14	–
OL_Y138F	0.48 ± 0.01 (0.01)	3.52 ± 0.02	0.52	50.72 ± 0.84 (0.72)
OL_S47A/H64F	0.03 ± 0.005 (0.0005)	2.01 ± 0.04	0.30	0.66 ± 0.02 (0.009)
OL_S47A/H64Q	1.27 ± 0.11 (0.02)	6.18 ± 0.03	0.92	27.30 ± 0.60 (0.39)
OL_H64F/Y138F	0.20 ± 0.05 (0.003)	0.25 ± 0.002	0.04	–
OL_H64Q/Y138F	0.04 ± 0.001 (0.001)	1.59 ± 0.07	0.24	11.63 ± 0.31 (0.16)
Aequorin				
AV_wt	5.43 ± 0.27 (1)	5.12 ± 0.09	1	17.01 ± 0.27 (1)
AV_S41A	1.87 ± 0.14 (0.34)	4.34 ± 0.17	0.85	3.20 ± 0.18 (0.19)
AV_H58F	0.02 ± 0.002 (0.004)	1.53 ± 0.05	0.30	0.11 ± 0.01 (0.006)
AV_H58Q	1.47 ± 0.04 (0.27)	3.63 ± 0.02	0.71	8.75 ± 0.21 (0.51)
AV_W108F	0.33 ± 0.03 (0.06)	0.52 ± 0.001	0.10	–
AV_Y132F	0.28 ± 0.002 (0.05)	2.62 ± 0.17	0.51	14.32 ± 0.44 (0.84)
AV_S41A/H58F	0.05 ± 0.003 (0.009)	1.75 ± 0.01	0.34	0.60 ± 0.03 (0.035)
AV_S41A/H58Q	1.20 ± 0.07 (0.22)	4.25 ± 0.05	0.83	8.38 ± 0.24 (0.49)
AV_H58F/Y132F	0.03 ± 0.002 (0.006)	0.14 ± 0.003	0.03	–
AV_H58Q/Y132F	0.08 ± 0.006 (0.02)	1.79 ± 0.01	0.35	6.07 ± 0.23 (0.36)

^a^ Maximal light intensity, specific bioluminescence activity, and Ca^2+^-independent luminescence of photoproteins were calculated using the average of five independent measurements. ^b^ Ratio of maximal light intensity of the mutant to that of the wild-type photoprotein. ^c^ Ratio of Ca^2+^-independent luminescence of the mutant to that of the wild-type photoprotein.

**Table 2:** Spectral properties of obelin and aequorin mutants.

Photoprotein	Absorption Spectral Maximum λ_max_ (nm)	Bioluminescence Spectral Maximum λ_shoulder_ (*)_/_λ_max_ (nm)	Fluorescence Spectral Maximum λ_shoulder_ (*)_/_λ_max_ (nm)
Obelin			
OL_wt	465	400 (0.17)/480	510
OL_S47A	465	400 (0.18)/480	420 (0.07)/508
OL_H64F	465	400 (0.05)/485	508
OL_H64Q	465	400 (0.08)/483	504
OL_W114F	345/450	400 (0.17)/485	410 (0.13)/506
OL_Y138F	455	400 (0.07)/487	420 (0.03)/512
OL_S47A/H64F	465	400 (0.05)/485	490
OL_S47A/H64Q	465	400 (0.09)/485	420 (0.05)/508
OL_H64F/Y138F	345/450	400 (0.07)/487	425 (0.37)/508
OL_H64Q/Y138F	455	400 (0.07)/492	425 (0.10)/510
Aequorin			
AV_wt	455	470	470
AV_S41A	435/470	465	470
AV_H58F	460	390 (0.04)/480	476
AV_H58Q	455	390 (0.04)/480	480
AV_W108F	345/445	470	470
AV_Y132F	445	395 (0.03)/475	395 (0.16)/480
AV_S41A/H58F	460	390 (0.03)/480	460
AV_S41A/H58Q	455	480	476
AV_H58F/Y132F	345/450	395 (0.06)/485	420/486 (0.71)
AV_H58Q/Y132F	445	480	420 (0.64)/480

* Contribution of a shoulder was estimated by averaging at least five independent measurements of bioluminescence and fluorescence spectra. Contribution was calculated as a ratio of intensity at a shoulder to that of the main peak.

**Table 3:** Kinetics of bioluminescent reactions of obelin and aequorin mutants.

Photoprotein	k_rise_	k_decay_	
		k_decay1_	k_decay2_
Obelin			
OL_WT	510.0 ± 5.0	40.00 ± 1.75 (0.66) ^a^	4.80 ± 0.05 (0.34)
OL_S47A	416.9 ± 3.5	44.88 ± 1.59 (0.23)	0.74 ± 0.002 (0.77)
OL_H64F	144.2 ± 4.2	0.0035 ± 0.0006	
OL_H64Q	236.8 ± 0.6	0.49 ± 0.07 (0.07)	0.13 ± 0.001 (0.93)
OL_W114F	406.7 ± 4.2	2.25 ± 0.02 (0.53)	0.21 ± 0.001 (0.47)
OL_Y138F	305.3 ± 1.4	13.7 ± 0.30 (0.80)	0.06 ± 0003 (0.20)
OL_S47A/H64F	158.1 ± 2.0	0.09 ± 0.01 (0.13)	0.003 ± 0.0006 (0.87)
OL_S47A/H64Q	214.5 ± 0.8	0.112 ± 0.0003	
OL_H64F/Y138F	218.8 ± 1.6	0.111 ± 0.0003	
OL_H64Q/Y138F	278.1 ± 2.2	4.10 ± 0.26 (0.18)	0.10 ± 0.001 (0.82)
Aequorin			
AV_WT	123.0 ± 1.0	0.81 ± 0.01	
AV_S41A	134.6 ± 1.4	0.36 ± 0.02 (0.43)	0.18 ± 0.01 (0.57)
AV_H58F	119.8 ± 0.4	0.26 ± 0.001 (0.52)	0.01 ± 0.0001 (0.48)
AV_H58Q	60.0 ± 0.2	0.35 ± 0.02 (0.46)	0.23 ± 0.01 (0.54)
AV_W108F	238.7 ± 3.8	0.88 ± 0.02 (0.43)	0.17 ± 0.01 (0.57)
AV_Y132F	32.4 ± 0.2	1.6 ± 0.21 (0.13)	0.05 ± 0.001 (0.87)
AV_S41A/H58F	51.4 ± 0.4	0.17 ± 0.01 (0.12)	0.013 ± 0.0001 (0.88)
AV_S41A/H58Q	70.1 ± 0.6	0.21 ± 0.002 (0.85)	0.10 ± 0.002 (0.15)
AV_H58F/Y132F	72.5 ± 0.6	0.87 ± 0.06 (0.07)	0.19 ± 0.001 (0.93)
AV_H58Q/Y132F	51.1 ± 0.8	0.21 ± 0.01 (0.35)	0.02 ± 0.001 (0.65)

^a^ Contribution of “fast” and “slow” components in decay kinetics was calculated as described in Materials and Methods Section 4.

**Table 4:** Data collection and refinement statistics.

	Obelin H64F Mutant (8C6O)
Wavelength, Å	0.9677
Resolution range, Å	38.76–2.2 (2.279–2.2)
Space group	P 61
Unit cell, Å °	77.51 77.51 180.22 90 90 120
Total reflections	161,998 (16,720)
Unique reflections	29,727 (3029)
Multiplicity	5.4 (5.5)
Completeness, %	95.9 (97.3)
Mean I/sigma (I)	8.73 (0.94)
Wilson B-factor, Å^2^	40.6
R-merge, %	16.4 (220.1)
R-meas, %	18.2 (242.7)
R-pim, %	7.6 (100)
CC1/2, %	99.5 (27.7)
CC*, %	99.9 (65.8)
Reflections used in refinement	29,719 (3028)
Reflections used for R-free	844 (60)
R-work, %	17.2 (30.2)
R-free, %	20.3 (30.2)
CC (work), %	96.2 (62.9)
CC (free), %	94.2 (62.0)
Number of non-hydrogen atoms	3446
macromolecules	3112
ligands	70
solvent	264
Protein residues	391
RMS (bonds), Å	0.010
RMS (angles), °	0.96
Ramachandran favored, %	98.2
Ramachandran allowed, %	1.8
Ramachandran outliers, %	0.00
Rotamer outliers, %	0.31
Clashscore	1.15
Average B-factor, Å^2^	43.2
macromolecules	42.9
ligands	44.3
solvent	47.4

Statistics for the highest resolution shell are shown in parentheses.

## Data Availability

All crystallographic coordinates and structure factors have been deposited in the PDB under the accession code 8C6O. All other data are available from the corresponding author on reasonable request.

## Supplementary Materials

The following supporting information can be downloaded at: https://www.mdpi.com/article/10.3390/ijms24076869/s1.
