# Supplementary material for: The Role of Tyr-His-Trp Triad and Water Molecule Near the N1-Atom of 2-Hydroperoxycoelenterazine in Bioluminescence of Hydromedusan Photoproteins: Structural and Mutagenesis Study
Source: Int J Mol Sci. 2023 Apr 6;24(7):6869. doi: 10.3390/ijms24076869 (PMC10095345; doi:10.3390/ijms24076869)
Supplement: Supplementary file 1 [file ijms-24-06869-s001.zip › ijms-2322262-supplementary.pdf]

Supplementary materials for

The Role of Tyr-His-Trp Triad and Water Molecule near the N1-atom of 2-hydroperoxycoelenterazine in Bioluminescence of Hydromedusan Photoproteins: Structural and Mutagenesis Study

Pavel V. Natashin[1], Ludmila P. Burakova[1,2], Margarita I. Kovaleva[3], Mikhail B. Shevtsov[3], Daria A. Dmitrieva[3], Elena V. Eremeeva[1,2], Svetlana V. Markova[1,2], Alexey V. Mishin[3], Valentin I. Borshchevskiy[3,4*] and Eugene S. Vysotski[1*]

[1] Photobiology Laboratory, Institute of Biophysics SB RAS, Federal Research Center "Krasnoyarsk Science Center SB RAS", Krasnoyarsk, Russia
[2] Institute of Fundamental Biology and Biotechnology, Siberian Federal University, Krasnoyarsk, Russia
[3] Research Center for Molecular Mechanisms of Aging and Age-Related Diseases, Moscow Institute of Physics and Technology, Dolgoprudny, Russia
[4] Joint Institute for Nuclear Research, Dubna, Russia

*Corresponding authors: Eugene Vysotski, e-mail <eugene_vysotski@ibp.ru>, tel. 7 (391) 2494430, fax 7 (391) 290-54-90; Valentin I. Borshchevskiy, e-mail <borshchevskiy.vi@phystech.edu> tel. 7 (964) 6328650, fax 7 (495) 408-68-69

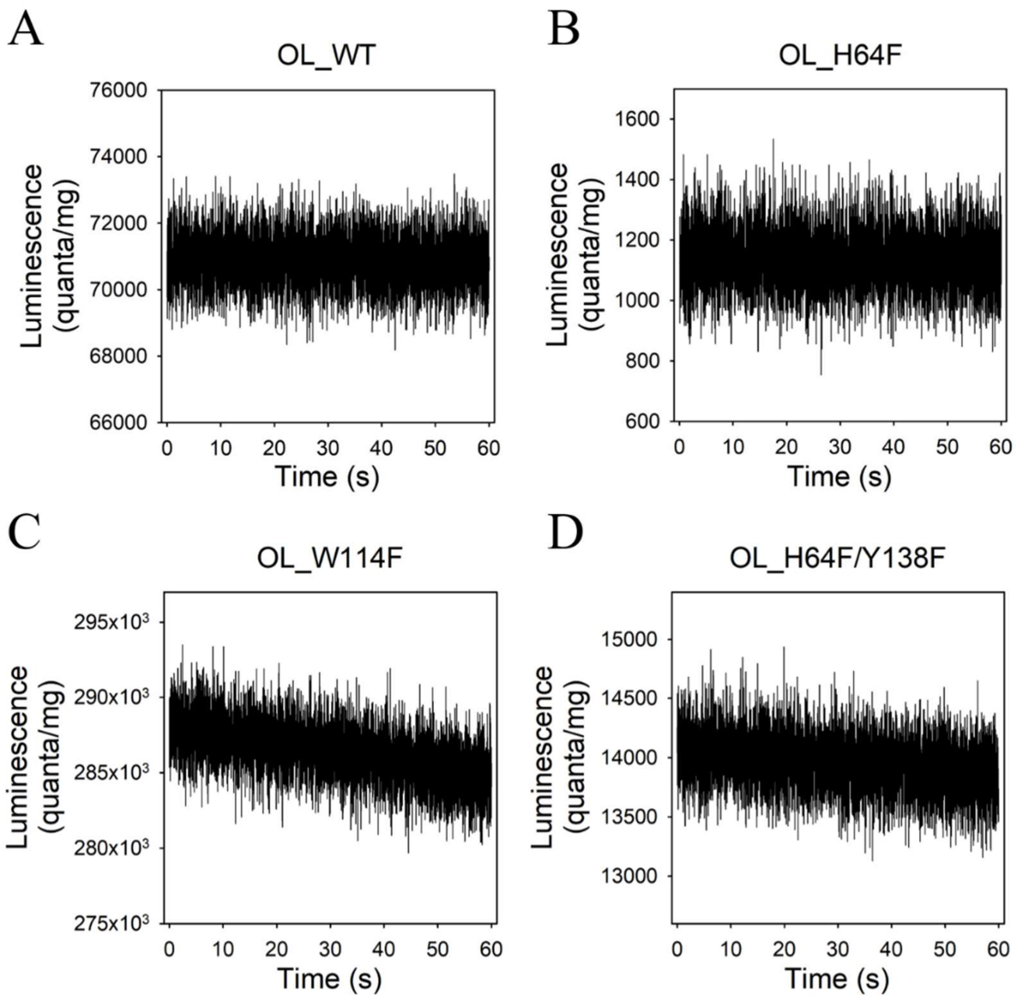

Figure S1. Ca^{2+}-independent luminescence of wild-type obelin (A), OL_H64F mutant (B), OL_W114F mutant (C) and OL_H64F/Y138F mutant (D).

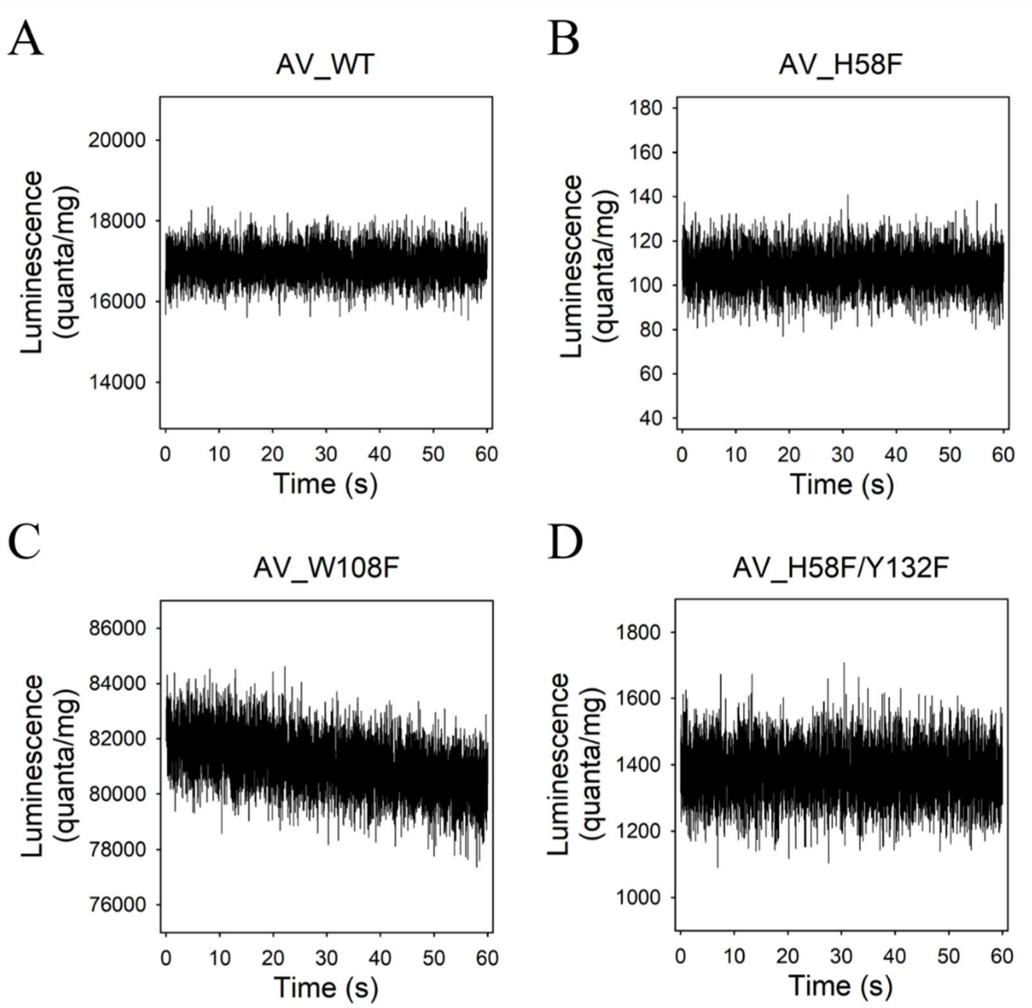

Figure S2. Ca^{2+}-independent luminescence of wild-type aequorin (A), AV_H58F mutant (B), AV_W108F mutant (C) and AV_H58F/Y132F mutant (D).
